# Developing and validating a nomogram based on skeletal muscle index and clinical scoring system for prediction of liver failure after hepatectomy

**DOI:** 10.3389/fonc.2023.1036921

**Published:** 2023-01-19

**Authors:** Cong Ding, Jianye Jia, Lei Han, Wei Zhou, Ziyan Liu, Genji Bai, Qian Wang

**Affiliations:** ^1^ Department of Radiology, The Affiliated Huaian No. 1 People’s Hospital of Nanjing Medical University, Huaian, Jiangsu, China; ^2^ Department of Radiology, The Affiliated Huaian Hospital of Xuzhou Medical University, Huaian, Jiangsu, China

**Keywords:** skeletal muscle index, post-hepatectomy liver failure, nomogram, liver, clinical score system

## Abstract

**Background and objectives:**

Hepatectomy is the preferred treatment for patients with liver tumors. Post-hepatectomy liver failure (PHLF) remains one of the most fatal postoperative complications. We aim to explore the risk factors of PHLF and create a nomogram for early prediction of PHLF.

**Methods:**

We retrospectively analyzed patients undergoing hepatectomy at the Affiliated Huaian No. 1 People’s Hospital of Nanjing Medical University between 2015 and 2022, and the patients were divided into training and internal validation cohorts at an 8:2 ratio randomly. The patients undergoing liver resection from the Affiliated Huaian Hospital of Xuzhou Medical University worked as external validation. Then, a nomogram was developed which was based on multivariate analyses to calculate the risk of PHLF. The area under the ROC curve (AUROC) and Hosmer -Lemeshow test was used to evaluate the prediction effect of the model.

**Results:**

A total of 421 eligible patients were included in our study. Four preoperative variables were identified after multivariate analysis as follows, ASA (American Society of Anesthesiologists) score, Child-Pugh score, SMI (Skeletal muscle index), and MELD (Model for end-stage liver disease) score as independent predictors of PHLF. The area under the ROC curve of the predictive model in the training, internal, and external validation cohorts were 0.89, 0.82, and 0.89. Hosmer -Lemeshow P values in the training, internal, and external validation cohorts were 0.91, 0.22, and 0.15. The Calibration curve confirmed that our nomogram prediction results were in accurate agreement with the actual occurrence of PHLF.

**Conclusion:**

We construct a nomogram to predict the grade B/C PHLF of ISGLS (International Study Group of Liver Surgery) in patients who underwent hepatic resection based on risk factors. This tool can provide a visual and accurate preoperative prediction of the grade B/C PHLF and guide the next step of clinical decision-making.

## Introduction

Although various techniques have been developed for treating liver tumors, hepatectomy remains the first line of treatment for liver tumors. With improvements in perioperative management and surgical techniques, liver resection is increasingly being performed for both benign and malignant diseases (1). PHLF remains one of the direst postoperative complications after liver resection and is one of the leading causes of perioperative mortality (2). Despite a comprehensive clinical understanding of the risk factors for PHLF, advances in surgical techniques, and improved prevention strategies to reduce the incidence of PHLF, the incidence of PHLF remains at around 8-32% (3, 4). It is of great importance to improve the prognosis of a patient’s morbidity or mortality by accurate preoperative prediction of PHLF.

Although the Child-Pugh score, MELD score, and ALBI score have been proposed to assess preoperative liver reserve function and predict post-hepatectomy complications, it has been suggested that their accuracy in predicting PHLF is limited and they do not take into account the nutritional status of the patients (5). Sarcopenia, a syndrome dominated by the loss of skeletal muscle mass and strength, has been reported as an important prognostic factor in predicting clinical outcomes for patients after cancer surgery (6). Several studies have now shown that sarcopenia assessed by the Skeletal Muscle Index (SMI), correlates with the prognosis of patients after hepatectomy (7–10). However, there is no comprehensive, convenient tool for predicting PHLF in patients undergoing hepatectomy which combined SMI and clinical scoring systems has been reported.

This study aimed to pick out predictors of PHLF before liver resection, including SMI and clinical scoring system, and to build a nomogram to predict the risk of PHLF in patients undergoing hepatectomy.

## Patients and methods

A total of 421 patients who had undergone hepatectomy from October 2015 to December 2021 at the Affiliated Huai’an No. 1 People’s Hospital of Nanjing Medical University and the Affiliated Huai’an Hospital of Xuzhou Medical University, were retrospectively enrolled in the study. Indications for liver resection include tumors without vascular invasion and Child-Pugh grade A/B patients, whereas Child-Pugh grade C is not indicated. Data including age, sex, height, weight, cirrhosis, tumor location, tumor size, type of operation, blood loss, AFP(α-Fetoprotein), ALB (Albumin), T-Bil (Total bilirubin), ALT (Aspartate aminotransferase), AST (Aspartate aminotransferase), GGT (γ-glutamyl transpeptidase), ALP (Alkaline phosphatase), CREA (Creatinine), PLT (Platelet), INR (International normalized ratio), and skeletal muscle index were analyzed. Patients were eligible if they had full clinical, radiologic, pathologic, and surgical perioperative data. Exclusion criteria were the following (1): previous locoregional (intrahepatic) treatments (2); unavailability or lack of clinical, radiological, pathologic, and surgical data. All procedures were following the ethical standards of the Ethical Committee of the Affiliated Huaian No. 1 Hospital of Nanjing Medical University (Ethical approval number: KY-2022-045-01)

### Liver resection

The hepatectomy involved both laparoscopic and open radical liver resection, including nonanatomic regional resection, segment resection, and lobe resection. Indications for liver resection include tumors without vascular invasion. Child-Pugh grade A and B, indocyanine green retention rate at 15 min of 20–30%, and the estimated remnant liver volume of≥35% are listed as prerequisites for resection. Locoregional therapy such as bland hepatic artery embolization, transarterial chemoembolization, and drug-eluting bead transarterial chemoembolization, were used for patients with advanced or unresectable disease who are not surgical candidates (11–13).

### Measurement of skeletal muscle mass

**Figure 1:**
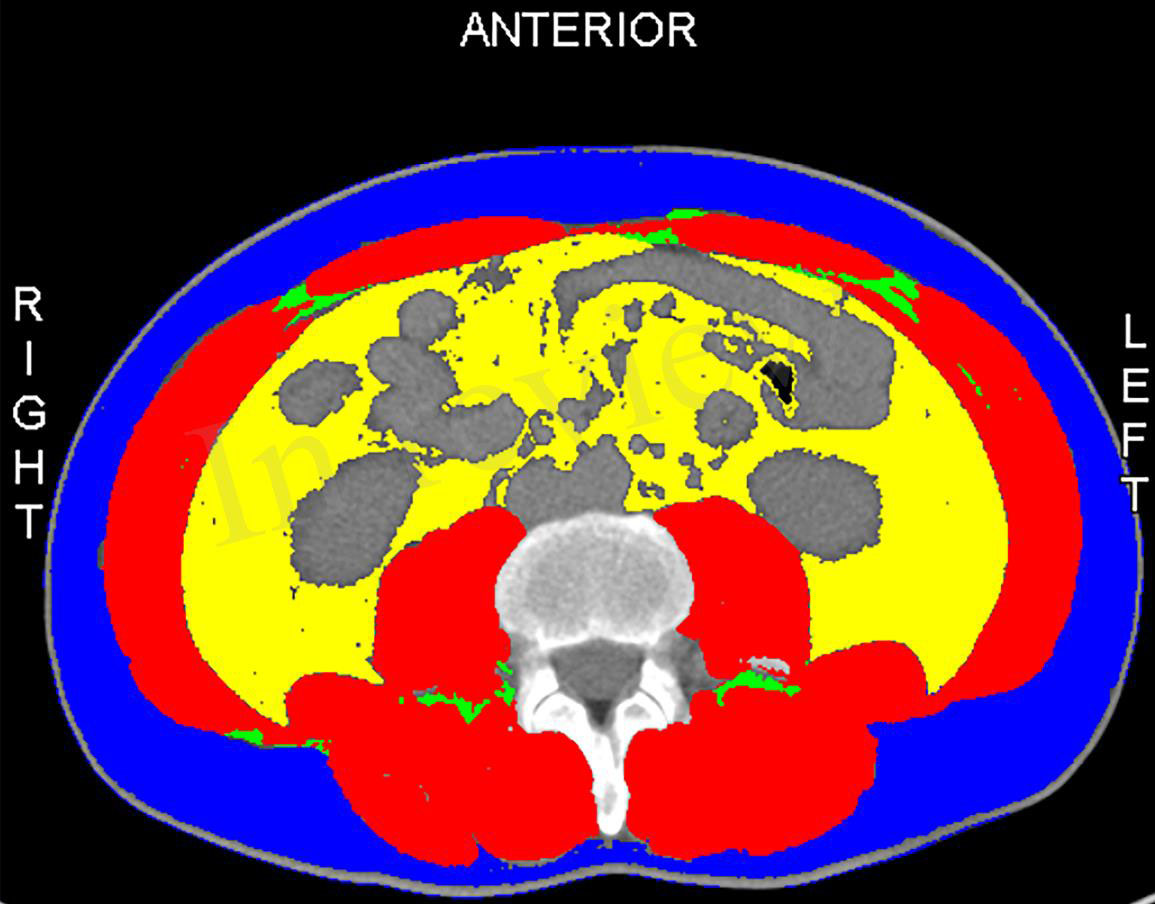
The skeletal muscles at the level of the third lumbar vertebra are painted in red.

All patients had preoperative computed tomography (CT). The skeletal muscle mass (cm^2^) was calculated for all patients by measuring the area of all skeletal muscles including the psoas, erector spinae, quadratus lumborum, transverse section of the lumbar spine, transverse abdominis, internal and external oblique muscles, and rectus abdominis at the third lumbar vertebra (L3) when both vertebral arches are visible. We normalized skeletal muscle area by height (m^2^) to calculate L3 SMI (**Figure 1**). The areas of skeletal muscle mass were measured by the sliceOmatic system (14). To minimize bias, two radiologists were unaware of clinical, biological, and other anthropometric data and were trained to calculate muscle area.

### Definitions of variables

PHLF is defined by the International Study Group of Liver Surgery (ISGLS) (3). The severity of PHLF is classified as A, B, and C according to the clinical management strategy. As patients with PHLF grade A do not need to change their clinical management and patients with grade B and C need to change their clinical management strategy or even opt for invasive treatment (15). So we focused only on PHLF grades B and C as outcomes because of their clinical importance.

### Calculation of clinical scores

The ALBI score formula was as follows:


$$\text{ALBI score }=\;\left(\text{log}10\text{ bilirubin }\left(\frac{\text{μmol}}{\text{L}}\right)\times \;0.66\right)+\;\left(\text{albumin }\left(\frac{\text{g}}{\text{L}}\right)\times \;\left(-0.085\right)\right)$$

(16).

The MELD score was calculated as follows:


MELD score =9.57 × ln(creatinine, , mg/dL) + 3.78 × ln (total bilirubin, mg/dL) + 11.2 × ln(INR) + 6.4

(17).

The APRI score was calculated as follows:


APRI score= ((AST (U/L)/ULN)/PLT count (109/L)) × 100

(18).

The FIB-4 score was calculated as follows:


FIB−4 score=AST (U/L) × age (years)/(platelet count (×109/L) × ALT (U/L)1/2)

(19).

The NLR defined as the absolute neutrophil count divided by the absolute lymphocyte count was calculated from a full blood test performed before surgery (20).

### Study design and statistical analysis

**Figure 2:**
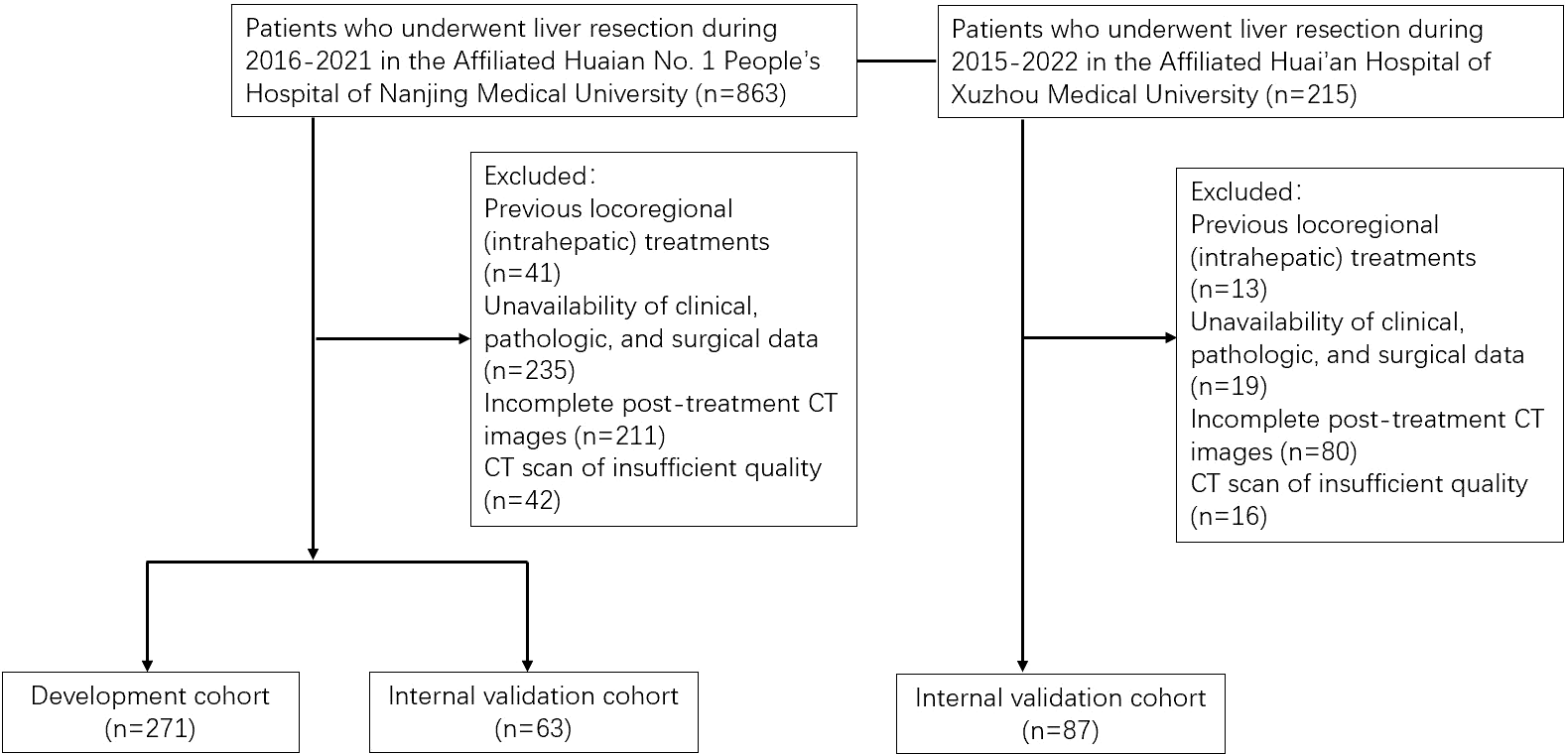
Flow chart for patient inclusion.

The flow chart of the study design is shown in **Figure 2**. The patients were divided into training and internal validation cohorts at an 8:2 ratio by random sampling. R (Version.3.5.3), and SPSS (Version.25.0, IBM, Armonk, NY, USA) were used to conduct analysis and draw plots. P< 0.05 was considered statistically significant. The distribution of clinical variables was compared using the analysis of variance for continuous variables and the Kruskal–Wallis H test for categorical variables. ROC curves are plotted using the pROC package and the area under the curve, sensitivity, specificity, and accuracy of the ROC curves are used to assess predictive performance. The DCA (decision curve analysis) curves are also plotted using the rmda package to analyze the value of the model. The calibration curve and Hosmer Lemeshow test were used to evaluate the predictive accuracy and conformity of the model.

## Results

### Patient characteristics

A total of 421 eligible patients were finally enrolled in this study, including 156 grade B/C PHLF patients(37.1%). Of them, 334 patients from the Affiliated Huaian No. 1 People’s Hospital of Nanjing Medical University were divided 8:2 into training and internal validation cohort randomly, and 87 patients from the Affiliated Huai’an Hospital of Xuzhou Medical University comprised the external validation cohort. Baseline demographic variables, laboratory tests, and intraoperative major events showed no significant differences between the training cohort, internal validation cohort, and external cohort, and the details are shown in **Table 1**.

**Table 1:** Clinicopathologic characteristics of study participants.

Variables	Training Cohort (n=271)	Internal validation Cohort (n=63)	External validation (n=87)	*P* value
Age (years)	59.48 ± 10.39	60.00 ± 10.80	60.36 ± 9.96	0.77
Sex, Male/Female	193/78	45/18	64/23	0.88
Height (m)	1.66 (1.60, 1.70)	1.66 (1.60, 1.70)	1.68 (1.60,1.71)	0.77
Weight (kg)	65.00 (57.50, 71.00)	62.00 (58.00,70.00)	65.00 (58.00,70.00)	0.81
Hepatitis				0.32
Yes	110	22	37	
No	161	41	50	
Cirrhosis				0.57
Yes	114	26	31	
No	157	37	56	
Fatty liver				0.64
Yes	259	61	85	
No	12	2	2	
Tumor location				0.57
Right lobe	200	43	66	
Left lobe	56	16	20	
Both the right and left lobe	15	4	1	
Tumor size (mm)	40 (27,60)	50 (30,64)	50.00 (31.5,80)	0.09
Tumor number				0.34
Single	251	56	77	
Multiple	20	7	10	
Type of resection				0.08
Minor resection	207	44	64	
Major resection	64	19	23	
Type of surgery				0.01
Laparotomy surgery	191	49	49	
Laparoscopic surgery	80	14	38	
Blood loss (mL)				0.21
<500	182	48	65	
≥500	89	15	22	
Operation time (h)	3.00 (2.00, 3.00)	2.50 (2.00,4.00)	2.63 (2.00.3.50)	0.06
AFP (ng/mL)	6.84 (2.79,203.14)	6.28 (2.95,195.00)	6.11 (3.00,138.89)	0.99
ALB (g/L)	41.6 (37.65,44.40)	41.3 (37.7,43.80)	42.15 (38.05,44.50)	0.8
T-Bil (μmol/L)	16.00 (10.90,25.75)	14.2 (10.50,25.30)	12.80 (9.70,16.88)	0.01
ALT (U/L)	30.00 (19.00,52.25)	26.70 (19.3,26.70)	24.00 (15.18,36.98)	0.04
AST (U/L)	31.00 (22.9,52.85)	30.90 (23.00,52.20)	28.35 (20.00,40.18)	0.11
GGT (U/L)	65.00 (30.00,150.00)	63.00 (32.00,142.00)	56.50 (27.50,122.50)	0.37
ALP (U/L)	95.00 (75.00,139.50)	91.00 (73.00,138.00)	89.00 (67.00,126.50)	0.42
CREA (μmol/L)	65.00 (54.40,75.00)	63.00 (32.00,142.00)	67.75 (56.23,77.05)	0.72
PLT (× 109/L)	153.00 (103.50,201.00)	167.00 (110.00,229.00)	178.50 (133.75,216.75)	0.04
INR	1.02 (0.97, 1.10)	1.01 (0.98, 1.06)	1.04 (0.98,1.07)	0.38
Neutrophil (× 109/L)	3.26 (2.49, 5.13)	3.79 (2.56, 6.01)	3.32 (2.59,5.05)	0.24
Lymphocyte (× 109/L)	1.3 (0.96, 1.69)	1.41 (0.92, 1.73)	1.44 (1.06,1.92)	0.06
ASA score				0.02
1	78	14	9	
2	145	35	59	
≥3	48	14	19	
Child-Pugh grade				0.28
Grade A	207	47	73	
Grade B	64	16	14	
Skeletal muscle index (cm^2^/m^2^)	48.61 (44.35, 55.65)	52.37 (45.09,55.38)	47.41 (43.60,52.14)	0.06
BMI (kg/m2)	23.53 (21.45,25.71)	23.74 (21.09,25.33)	23.52 (21.72,25.41)	0.75
MELD score	7.23 (6.43,8.70)	7.17 (6.43,8.67)	6.98 (6.43,7.59)	0.54
NLR	2.51 (1.74,3.99)	2.87 (1.91,5.67)	2.60 (1.59.3.80)	0.21
FIB-4 score	2.58 (1.55,4.44)	2.30 (1.46,3.57)	2.06 (1.45,3.01)	0.04
ALBI score	-2.75 (-3.02,-2.29)	-2.81 (-3.02,-2.27)	-2.84 (-3.05,-2.46)	0.25
APRI score	0.64 (0.31,1.28)	0.49 (0.31,1.11)	0.39 (0.26,0.73)	0.01

AFP, α-Fetoprotein; ALB, Albumin; T-Bil, Total bilirubin; ALT, Aspartate aminotransferase; AST, Aspartate aminotransferase; GGT, γ-glutamyl transpeptidase; ALP, Alkaline phosphatase; CREA, Creatinine PLT, Platelet; INR, International normalized ratio; ASA score, American Society of Anesthesiologists score; BMI, Body mass index; MELD score, Model for end-stage liver disease score; NLR, neutrophil-to-lymphocyte ratio; ALBI, Albumin–bilirubin; APRI, Aspartate aminotransferase to platelet ratio index.

### Nomogram for PHLF grade B–C

**Figure 3:**
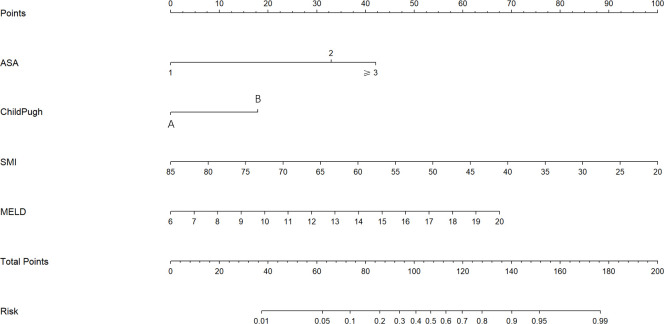
The nomogram for the prediction of PHLF. The nomogram was established based on the training cohort. PHLF, post-hepatectomy liver failure.

**Figure 4:**
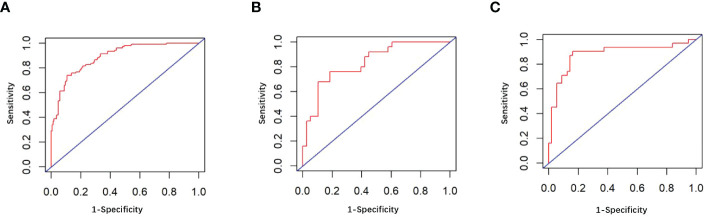
**(A)** The ROC curve in the training cohort; **(B)** The ROC curve in the internal validation cohort; **(C)** The ROC curve in the external validation cohort.

**Figure 5:**
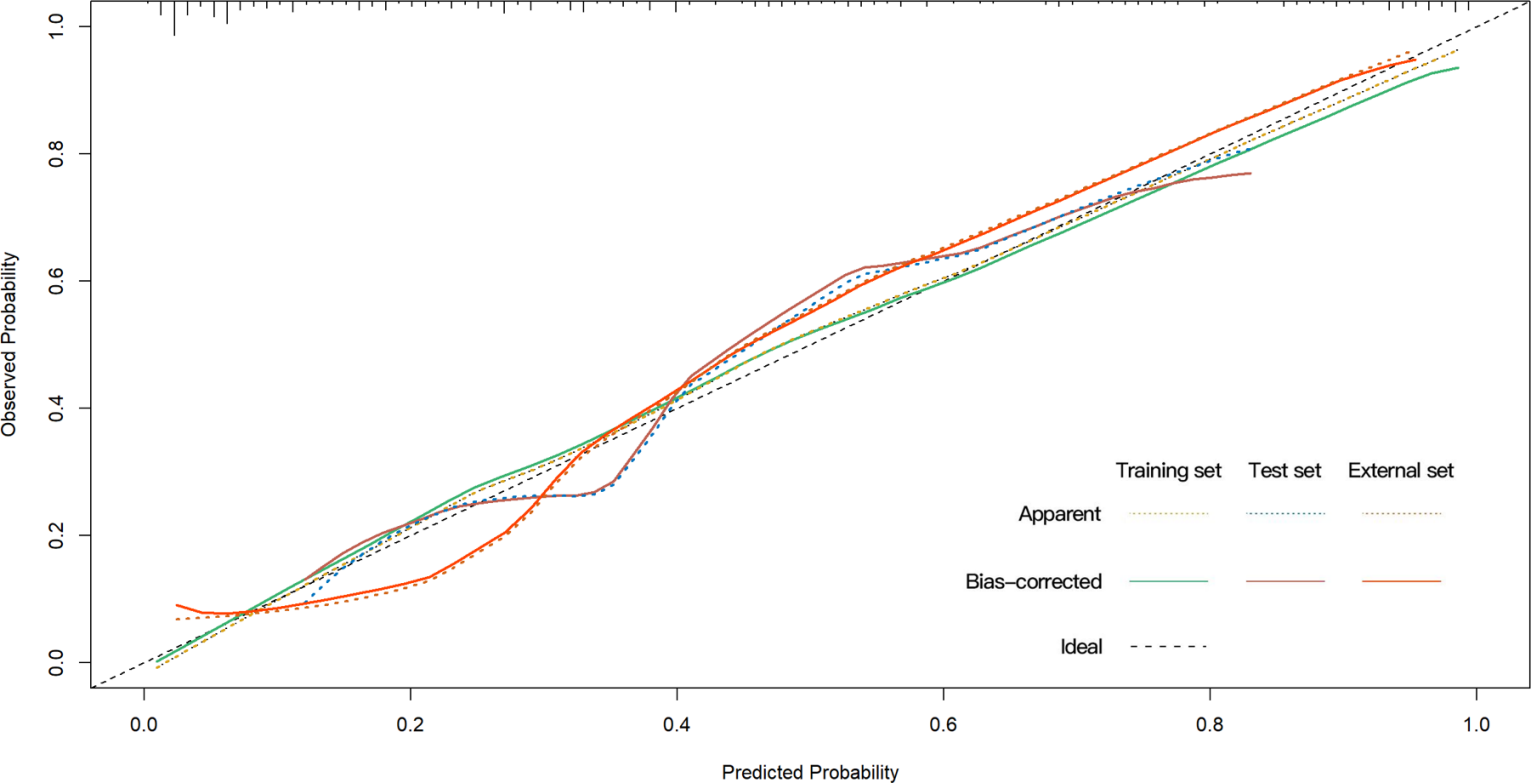
Calibration curves for the prediction of PHLF. PHLF, post-hepatectomy liver failure.

**Figure 6:**
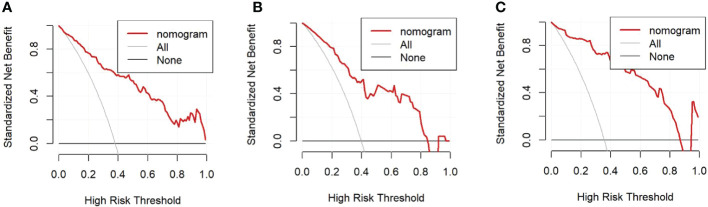
**(A)**. Decision curve in the training cohort; **(B)**. Decision curve in the internal validation cohort; **(C)**. Decision curve in the external validation cohort.

The univariate analysis suggested that the ALBI score, ASA score, SMI, Child-Pugh score, and MELD score were potential risk factors for PHLF in the training cohort. The multivariate analysis showed that the ASA score, SMI, Child-Pugh score, and MELD score were independent risk factors of PHLF (**Table 2**). We establish a nomogram to predict PHLF based on these independent risk factors in the training cohort (**Figure 3**), which can predict the risk of PHLF precisely, and the AUROC was 0.89 (**Figure 4A**). The calibration curves showed that the predicted risk of PHLF was in excellent agreement with the actual risk of PHLF (**Figure 5**). Hosmer -Lemeshow P value in the training cohort was 0.91. The decision curve shows that the nomogram has a perfect net benefit in the training cohort. (**Figure 6A**)

**Table 2:** Multivariable logistic regression analyses of grade B/C.

PHLF in the training cohort.Variables	Statistical data		
	OR	95%CI	*P*-value
SMI	-0.106	-0.153- -0.063	0.001
MELD score	0.381	0.119 - 0.693	0.001
ASA score			
1			0.001
2	2.302	1.262 -3.561	0.001
≥3	2.925	1.653 -4.365	0.001
Child-Pugh grade	1.444	0.226 -2.726	0.022

OR, odds ratio; 95%CI, 95% confidence interval; SMI, Skeletal muscle mass; MELD score, Model for end-stage liver disease score; ASA score, American Society of Anesthesiologists score.

### Validation of the nomogram

The value of AUROC in the internal and external validation cohorts are 0.82 and 0.89 (**Figures 4B, C**). The calibration curves showed that the predicted risk of PHLF was strong in consistency with the actual risk of PHLF. Hosmer -Lemeshow P values in the internal and external validation cohort were 0.22, and 0.15. The decision curve shows that the nomogram gets the ideal net benefit in the internal and external validation cohort (**Figures 6B, C**).

## Discussion

PHLF is one of the most acute complications in patients who suffered hepatectomy (2). Therefore, a nomogram was developed and validated for preoperative prediction of PHLF, based on data from multicentre cohorts of patients with liver tumors who underwent hepatectomy. The nomogram shows excellent performance in both the training, internal, and external validation cohorts, with AUROC of 0.89, 0.82, and 0.89, respectively. The model has been externally validated showing good discriminability and calibration and can individually assess the risk of PHLF precisely.

Patients with low SMI are more likely to develop PHLF than those with high SMI. The reason for this may be that SMI reflects the nutritional status of the patient and is an indirect marker of the patient’s frailty, defined as a condition that can reflect a reduction in the patient’s physiological reserves and therefore may affect the outcome after surgery (21, 22). ASA scores are now commonly used to assess a patient’s holistic composite fitness before anesthesia, and we found that patients with high ASA grades had a higher risk, which is consistent with previous studies (23). Child-Pugh scores, although having the disadvantage of being subjective, is still the most widely used clinical method for preoperative assessment of liver reserve function. The present study found that patients with Child-Pugh B and C patients were at high risk, which is similar to the findings of Xu et al. (24). The MELD score is widely used to assess the risk of end-stage liver disease and is also thought to predict PHLF and mortality after hepatectomy in patients with hepatocellular carcinoma, which is consistent with our findings (24–26).

Several previous studies have also attempted to predict the occurrence of PHLF. The model constructed by Lei was based on age, gender, T-bil, PT, and CSPH(clinically significant portal hypertension), and the model only included patients with hepatitis B-associated liver cancer (5). The patients included in our study not only included patients with hepatitis B-associated liver cancer, demonstrating wider clinical applicability. CSPH may cause injury to the patient, which is examined endoscopically. The nomogram created by XU based on Child-Pugh grades which is similar to our study demonstrates the advantage of the Child-Pugh grades in predicting PHLF. However, it only included patients with HCC (diameter ≥ 10 cm), and its inclusion of intraoperative blood loss could not provide an accurate prediction of PHLF preoperatively. The nomogram constructed by Fang incorporates cirrhosis, but its assessment of cirrhosis is based on imaging evaluation rather than histologically confirmed (27). Therefore it is less accurate for the diagnosis of early occult cirrhosis, while its inclusion of intraoperative blood loss, like that of Xu, makes it difficult to assess PHLF preoperatively. Meanwhile, its inclusion of tumor size is subjective and without a stated criterion for judging (28). Chin developed a model to predict PHLF, but the model included patients with colorectal cancer with liver metastases who received preoperative neoadjuvant chemotherapy, and the model was not validated, so it is uncertain whether it can predict PHLF accurately in other data sets (29). The present study has several strengths compared to the studies mentioned above. Firstly, our study is a multicentre study. Secondly, the nomogram was able to accurately predict the risk of PHLF in patients with liver tumors using only four clinical factors. Finally, the accuracy and stability of our model were excellent in both internal and external validation, with AUC values of 0. 82 and 0. 89, respectively, and we further validated the model for clinical application using DCA. This nomogram can provide an accurate probability of PHLF in patients with liver tumors and help clinicians manage and make decisions for patients who require early intervention to prolong survival.

Although the present study has these strengths, the following shortcomings remain. First, the present study was a retrospective study that excluded individual cases with incomplete data, which may lead to selectivity bias, which requires more prospective studies for further validation. Second, the model in the present study included fewer risk factors. Therefore, more risk factors should be included in the next validation studies to further improve the predictive power of the model.

In summary, by combining four essential preoperative parameters(SMI, MELD score, ASA score, Child-Pugh score), an individualized nomogram was established to predict the grade B/C PHLF of ISGLS in patients who underwent hepatectomy. The nomogram could serve as a convenient, accurate, objective, non-invasive, intuitive tool to facilitate clinical decisions.

## Data availability statement

The datasets presented in this study can be found in online repositories. The names of the repository/repositories and accession number(s) can be found in the article/**Supplementary Material.**

## Ethics statement

The studies involving human participants were reviewed and approved by the Ethical Committee of the Affiliated Huaian No. 1 Hospital of Nanjing Medical University. Written informed consent for participation was not required for this study in accordance with the national legislation and the institutional requirements. Written informed consent was obtained from the individual(s) for the publication of any potentially identifiable images or data included in this article.

## Author contributions

Design of the work: CD and JJ. Acquisition and analysis of data: LH, WZ, QW, and ZL. Manuscript writing: CD and GB. Final approval of manuscript: GB. Administrative support: GB. All authors contributed to the article and approved the submitted version.
